# An exploration of the views of paramedics regarding airway management

**DOI:** 10.1186/s13049-016-0243-2

**Published:** 2016-04-27

**Authors:** Janet Brandling, Megan Rhys, Matthew Thomas, Sarah Voss, Sian Emma Davies, Jonathan Benger

**Affiliations:** Department of Health and Applied Sciences, University of the West of England, Bristol, UK; Research Paramedic, South Western Ambulance Service NHS Foundation Trust, Exeter, UK; Anaesthesia and Critical Care, University Hospitals Bristol NHS Foundation Trust, Bristol, BS2 8HW UK; Emergency Care, University of the West of England, Bristol, UK; University Hospitals Bristol NHS Foundation Trust, Bristol, UK

**Keywords:** Airway management, Cardiac arrest, Paramedics

## Abstract

**Background:**

Paramedics are a skilled group of clinicians with expertise in airway management. Our research group has completed a trial comparing supraglottic airway devices with tracheal intubation during out of hospital cardiac arrest. This is a contentious topic amongst paramedics in the United Kingdom (UK). We explored the customs and beliefs of UK paramedics in relation to airway management, and whether tracheal intubation contributes to and sustains paramedic professional identity.

**Methods:**

The study took place within South Western Ambulance Service NHS Foundation Trust. We used a qualitative approach, conducting interviews and focus groups with paramedics. The themes arising from interviews were discussed in focus groups, developing a deeper understanding and providing insight and recommendations for future research and policy. Purposive sampling accounted for differing training and for participation in the main trial. There were 17interviews and five focus groups with a further 17 participants. Data saturation was achieved.

**Results:**

Four domains were identified. Pride - The ability to use a life-saving skill in austere conditions. Utility - Different training routes and experience have led to different attitudes towards airway management. Inconsistent expectations - Paramedics felt that there were different perceptions of their abilities amongst hospital staff and the general public. Professionalization - Debate over airway management is not founded on good evidence.

**Conclusion:**

We have demonstrated that UK paramedics have a wide range of views regarding airway management, and that these are based on evidence and experience rather than dogma. Airway management contributes to paramedics’ professional identity, but is not reliant on this.

## Background

Out of hospital cardiac arrest (OHCA) is a common medical emergency with a very poor prognosis: there are 118 OHCAs per 100,000 population per annum [1], and approximately 7 % survive to hospital discharge [2].

Our research group has recently completed a research study (REVIVE-Airways) that assesses the feasibility of completing a definitive randomised trial to compare a supraglottic airway device with tracheal intubation during OHCA [3].

The skill of tracheal intubation is a contentious topic amongst paramedics [4]. Recommendations from the Airway Working Group of the Joint Royal Colleges Ambulance Liaison Committee (JRCALC) conclude that: “The weight of evidence suggests that prehospital intubation without the use of drugs can worsen patient outcome. Supraglottic airway devices (SADs) have been shown to be safe and effective devices in elective and emergency hospital procedures … Ambulance trusts should be encouraged to adopt and use these devices as an alternative to tracheal intubation [5]”. This recommendation led to a detailed response from the College of paramedics, defending the practice of pre-hospital tracheal intubation, which is viewed by many paramedics as an essential and valuable skill [6]. This skill, among others contributes to the individual, interpersonal and societal-institutional paramedic professional identity [7]. Para-medicine is a developing discipline [8, 9], and although it is assimilated into the allied health professions through the regulatory body of the HCPC it continues to differentiate itself. It’s professionalization project and subsequent status is dependent upon how it is viewed at street level and by managers and educators within the profession as well as other professions outside [10, 11].

Whilst it proved possible to engage the required number of paramedics in our feasibility study, the majority of eligible paramedics within South Western Ambulance Service NHS Foundation Trust chose not to participate, and the strength of feeling regarding airway management in pre-hospital cardiac arrest has been apparent. We therefore undertook this companion qualitative study to explore paramedic views on resuscitation research and airway management and the reasons why paramedics did, or did not, take part in our feasibility study.

Literature review identified only two previous studies of paramedics’ perceptions of airway management and tracheal intubation: these were from North America, and not specific to cardiac arrest [12, 13].

The aim of this study was to understand paramedic views and behaviours in relation to pre-hospital airway management and resuscitation. Our objectives explored the following areas:

1. The existing customs and beliefs influencing pre-hospital intubation and resuscitation by paramedics in the South West of England;
2. Whether intubation skills contribute to and sustain the professional identity of the paramedic, and how this might change and adapt in future.

## Methods

A two level qualitative approach was used, conducting interviews and focus groups with paramedics. The interviews identified issues arising for paramedics as individuals and as a professional group. Focus groups were used to discuss the themes arising from the interview data in depth, developing a deeper understanding but also providing insight and recommendations for future research and policy. The study followed our feasibility study, REVIVE-Airways [3]. Both took place within South Western Ambulance Service NHS Foundation Trust. This research was funded by the Resuscitation Council UK and sponsored by the University of the West of England, Bristol. The funder and sponsor took no part in the design, conduct or reporting of the study. Since participants were NHS staff, NHS ethics committee approval was not required. However the project was reviewed and governance provided by the research ethics committee of the University of the West of England, Bristol.

It is known that paramedics have been trained either through a vocational pathway accredited by the Institute of Health Care Development (IHCD), and more recently paramedics have been educated to degree level by Higher Education Institutes (HEI). This may impact on paramedic beliefs and customs. Furthermore, some paramedics have expressed strong opinions on this topic in relation to REVIVE-Airways. Some were ineligible to take part in our feasibility study, whilst some declined to do so. Therefore a purposive sampling strategy, taking account of these factors, was used. Invitations were sent to paramedics who fitted the sampling categories; thereafter a snowballing technique was used.

Interviews and focus groups were held either within the workplace or on a University campus, and were conducted by an independent qualitative researcher. This researcher was advised on technical issues by a physician and a paramedic, both of whom had been on the study management team of the original trial. The interviews lasted between 11 and 50 min, while focus groups lasted 60–90 min. All were audio-recorded and recordings transcribed verbatim. Participant contribution was acknowledged with a gift voucher to the value of £10.

Thematic analysis was used and performed by an independent qualitative researcher. This was done in 2 stages:

A. After interviews, for presentation and discussion at the focus groups;
B. After focus groups, for final reporting.

A well-established iterative process of thematic analysis was used by the researcher to analyse each verbatim transcript [14]. This included:

1. Familiarisation with the data: reading and re-reading transcripts
2. Generating initial codes: noting codes of interesting and pertinent ideas
3. Searching for themes: systematically organising these recurrent ideas with extracts of text
4. Reviewing themes: checking themes are meaningful and relate to the text
5. Defining and naming themes: summarising the narrative with clear definitions
6. Producing the report: using extracts of data to exemplify the themes

To supplement and triangulate the analysis, paramedics and prehospital clinicians from the study team reviewed and commented on the themes presented independently.

Confidentiality, the right to refuse specific questions and withdraw were ensured via the consent process. A wide variety of paramedics felt able to participate, rather than just those with the strongest opinions or vested interests [15].

## Results

There were 34 study participants, with 17 paramedic interviews followed by five focus groups involving a further 17 individuals. Data saturation was achieved, with no new themes were emerging from the data. The available population was also limited, and so recruitment was ceased [16].

The characteristics of the 34 participants, in terms of their training and participation in REVIVE-Airways, are summarised in Tables 1 and 2.

**Table 1:** Interview participants

Interview participants	IHCD trained		HEI trained		Total
	REVIVE	Excluded from or declined REVIVE	REVIVE	Excluded from or declined REVIVE	
Number	4	4	5	4	17

**Table 2:** Focus group participants

Focus group participants	IHCD trained		HEI trained		Total
	REVIVE	Excluded from or declined REVIVE	REVIVE	Excluded from or declined REVIVE	
Number	5	7	3	2	17

It is clear from the accounts given by paramedics in this study that there are strong themes arising about paramedic identity, and how this relates to patient safety during the course of their work.

**Fig. 1:**
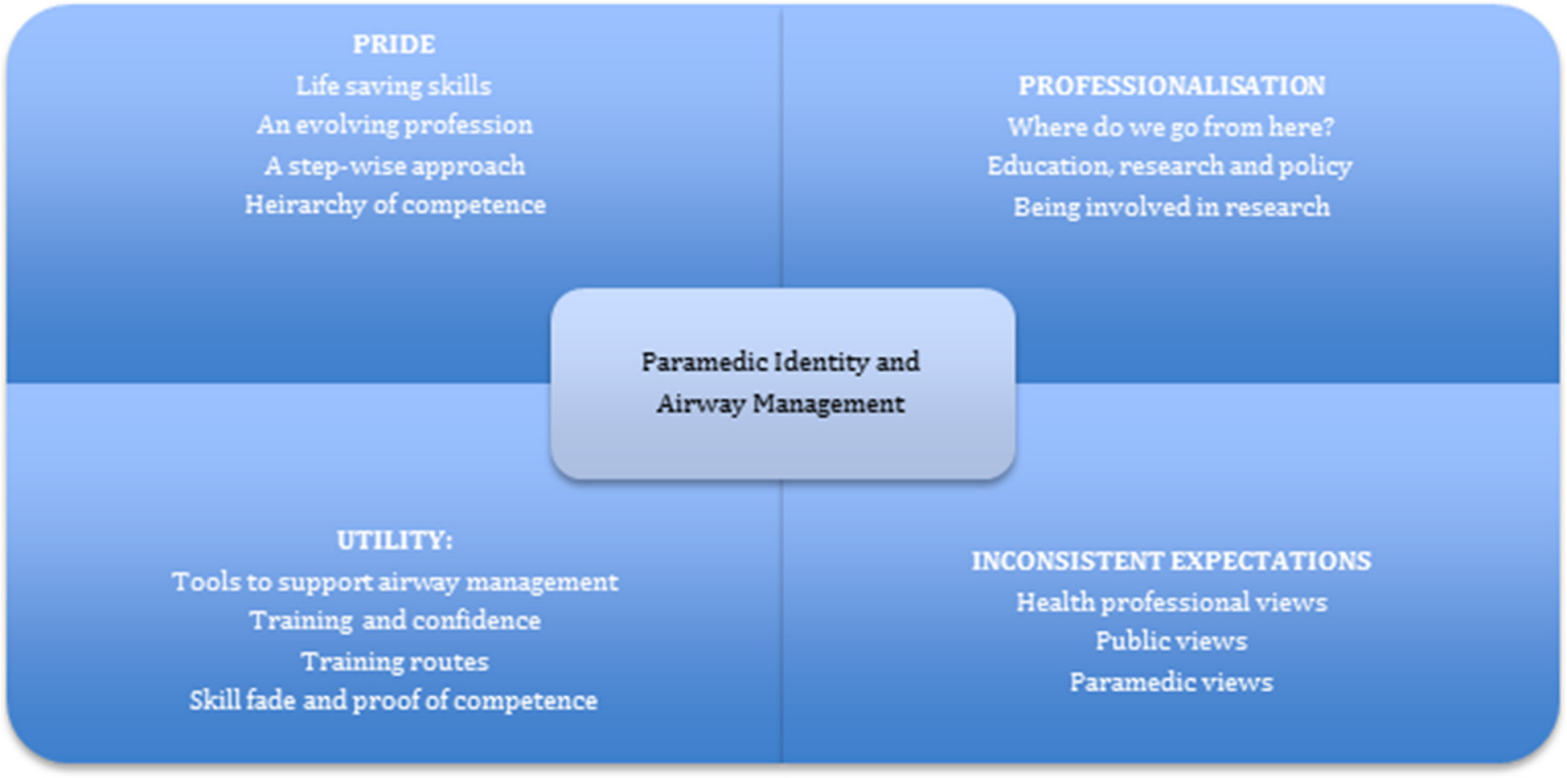
Paramedic Domains

Figure 1 illustrates the four domains into which paramedic identity was divided to discuss their opinions of airway management in practice.

### Domain 1: pride

#### Life saving skills

The debate about retaining the skill of intubation was contentious for this group, and many of the paramedics immediately defended their practice of intubation, suggesting they would be extremely unhappy to relinquish it. This quote exemplifies this viewpoint.

*“I will defend that skill to my dying day, really, because I think it’s still something we should have in our arsenal”* (FOCUS GROUP4)

This view appeared to be based on concern for patient safety; the possibility of not being able to respond adequately to life threatening events. Intubation is a technique that the paramedic can trust in case of aspiration and patient transport.

#### A step-wise approach

The utility and necessity of resuscitation skills was discussed in depth. The paramedics provided consistent narratives about the practice and step-wise manner of resuscitation and intubation. They expressed value for supraglottic devices as a component of this approach, with progression to intubation when necessary.

*“… I work on my own and I am going to have to do ALS until the troops arrive… I found the i-gel brilliant actually … and that was much better than just sort of an OP airway bag and mask …But I still agree that there is a place for intubation.”* (FOCUS GROUP2)

Good resuscitation was in part due to the guidance issued in recent years. Although intubation is considered a treatment of last resort, its loss would be to remove a core skill, disabling paramedic ability to respond in absolute necessity. The geographic distant from hospital, having patients being trapped in awkward situations, with family members, pets and other observers involved and difficult retrieval circumstances were presented as contributory factors. Patients often had full stomachs, already soiled airways, injuries and co-morbidities; all indications to move immediately to intubation as an airway management technique.

*“…because we are in an environment where the patients are not fasted, often have an airway full of detritus, whether it’s vomit or fluid or a piece of pork pie and it is one of the last ditch adjuncts that we can use to maintain that airway and there’s only us… no doctors, no anaesthetists, just you, so it is absolutely a key skill.”* (FOCUS GROUP2)

This clear narrative was a primary response to interview questions and reflects protocols used in paramedicine but as interviews progressed it began to be contested. Some respondents suggested a move from universal skills to a hierarchy, where specialist skills are targeted when necessary, and are performed by individuals experienced in that technique.

#### Hierarchy of competence

**Fig. 2:**
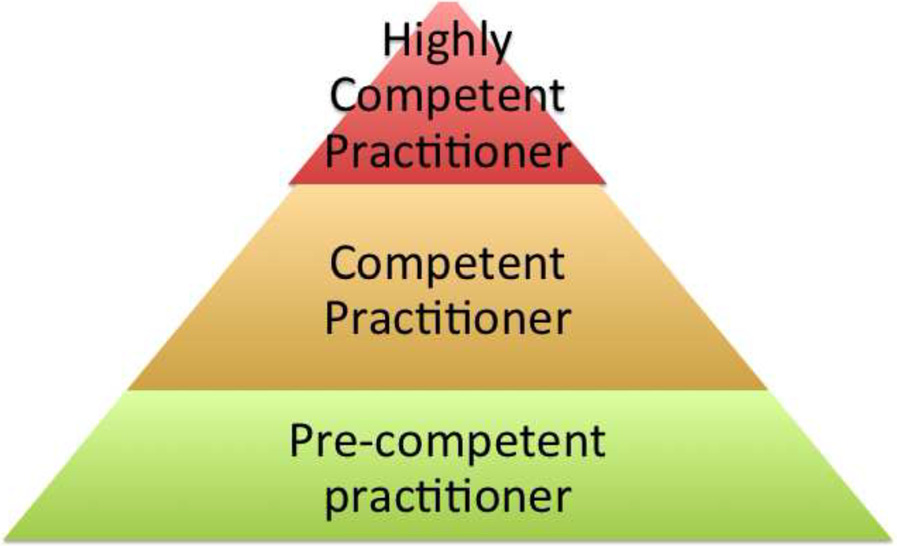
Hierarchy of Competence

A hierarchal view became apparent (Fig. 2), with a pyramid of specialism and with it the ability to use intubation skills. This was situated firmly in the top levels.

At the bottom of this pyramid inexperienced practitioners who have yet to develop competence require easy to use airway management techniques, while at the top the most highly specialist practitioners retain the highly desirable skill of intubation. Between these levels fall competent practitioners, and many respondents saw themselves in this zone. However this is clearly a contested zone, with variable views of competency and deservedness.

Participants wondered if it would work in practice, with a competent specialist in attendance:

*“I think if only certain paramedics had it then you’d end up with situations where you needed it on scene but you couldn’t get somebody there to do it… So I think it needs to be a skill for everybody otherwise when you need it you won’t have it.”* (FOCUS GROUP1)

Overall there was a strong sense of pride in resuscitation skills during the paramedic interviews and focus groups. Although there were several participants who questioned this view, most of the respondents wished to keep the skill and strongly defended tracheal intubation as part of paramedic identity.

### Domain 2: utility

#### Tools to support airway management

This theme represents the practicalities of skilled airway management and the mechanisms that support good practice. The participants discussed recently acquired tools to support airway management, such as intubating bougies and capnography. These tools enhanced their ability to successfully maintain the airway and intubate.

*I find that we’ve actually got more equipment now. They’ve got introducers and bougies and people are starting to use them and intubations are becoming more successful because they’ve used the equipment correctly… It takes a lot of the guesswork out of it …”* (FOCUS GROUP2)

#### Training routes

The participants reflected upon training and variable views resulted from differences between the IHCD training pathway and the HEI route. Intubation as a core skill seemed to depend upon this:

*“…experientially older staff, is they often see it as a core skill of a paramedic whereas perhaps newer people who have come through the university route where in the last few years intubation hasn’t been quite pushed as much as it was when some of us older ones went through, it’s not as important.”* (FOCUS GROUP5)

During IHCD training paramedics were required to complete 25 intubations in a hospital environment under the tutelage of an anaesthetist. Once completed, they were certificated competent. The HEI route does not have a similar process, and was suggested to be an inferior training mechanism.

The repetitive rehearsal in IHCD training was seen as providing skill confidence. For some this was a motor skill; once learned, never forgotten.

*“…intubation really is a piece of cake… you know. If you’ve got the equipment and you keep it up together it is second nature*.” (FOCUS GROUP4)

HEI trained paramedics, on the other hand, were often more sanguine about airway management skills, finding the supraglottic airway devices sufficient in most instances. Nevertheless they rarely wanted to relinquish intubation altogether. It was referred to as the ‘gold standard’, as if the pinnacle of practice. After the initial discussion of intubation paramedic views of their own competence, in relation to skill fade, were raised.

#### Skill fade, confidence and proof of competence

Participants discussed statutory, mandatory and personally acquired training subsequent to basic training. Many talked of ‘skill fade’, and noted a lack of confidence in themselves or colleagues. Some admitted they didn’t always feel confident about intubation.

*“..if you are not well versed at doing it, faffing around trying to do this intubation, you know, I find it’s difficult at times…I’d just like to be more trained in it and keep the skills up so I’d be more efficient at it.”* (FOCUS GROUP2)

Participants also discussed the increased need to develop skills such as assessment, diagnosis and drug administration, which relates to a greater proportion of their work, rather than focus so directly on resuscitation.

### Domain 3: inconsistent expectations

#### Health professional views

Expectations of paramedic skill were thought to be different according to observer. Some were cynical about other health professional’s view of their abilities, and felt that they were at risk of being criticised by other professions who lacked insight into the complexity of their work. Some had encountered frosty or critical receptions by other health professionals in emergency departments because they had brought in people who did not need emergency care.

*“I don’t think they have the comprehension of trying to manage somebody’s airway when they’re trapped between the bath and a toilet, or they’re at the bottom of a ditch and it’s dark and it’s blowing a hooly and you can’t see. There isn’t the comprehension at all*.” (FOCUS GROUP2)

In some cases they felt that their skill and training counted for little and they were viewed as little more than ‘ambulance drivers’. Specifically, it was suggested that some health professionals think retention of intubation skills is related to paramedic ego rather than necessity.

#### Public views

The public, on the other hand, were considered to hold paramedics in high regard, and have high expectations.

*“I think we’re… generally we’re held in really high esteem by the public and normally most people think we are like doctors…”* (FOCUS GROUP3)

Participants felt that members of the public did not distinguish between paramedics and emergency care assistants, and in an emergency situation would expect a paramedic response, with personnel able to utilise extended skills regardless of training and capacity.

#### Paramedic views

Paramedics demonstrated conflicting views of their own profession, largely related to the training route they had taken. IHCD trained paramedics valued the ‘on-the-road’ practical experience compared to HEI trained practitioners, who prioritised theory. On the other hand HEI practitioners considered themselves autonomous, able to critique practice rather than slavishly following protocol and guidelines. This issue represented a considerable, although not acrimonious, divide between the two groups.

*“And we used to take people to hospital because we were scared if we left them at home something might happen. But now we have the knowledge and we know why we’re leaving them at home.”* (FOCUS GROUP2)

### Domain 4: professionalization

#### Where do we go from here?

Discussions of paramedic identity led to broader consideration of the profession’s future. Only some paramedics had considered this in detail. The recommendations made by the Airway Working Group of the Joint Royal Colleges Ambulance Liaison Committee [2], were viewed as unwarranted criticism and provoked discussion. Paramedics robustly defended their profession and position.

*“Contrary to popular belief paramedics are an intelligent group of people who will listen to argument, who will consider research in the right light and will come to a reasonable conclusion, I think, but I think it’s when it’s forced on you… that’s when you sort of get the kick back.”* (FOCUS GROUP3)

This appears to be specifically directed towards doctors, where the efficacy of intubation in the pre-hospital environment was raised. They noted that the debate had served to galvanize their professional group, stimulating a desire to lead the direction of education, research and practice.

## Discussion

Prehospital airway management, and tracheal intubation in particular, has studies both supporting it and suggesting it can cause harm [4, 17]. Our study was the first UK research to qualitatively assess paramedic airway management.

Participants were largely reluctant to relinquish intubation skills in favour of other devices, even if tracheal intubation remained available to a smaller group of more highly-trained paramedics. This is similar to the findings from a North American study of pre-hospital intubation, where it was believed strongly that paramedics should retain this skill [13]. In our study this view was justified by perceived clinical need, and the challenges of delivering excellent care in the out-of-hospital environment, and is consistent with previous research from North America looking at the skills most highly ranked by paramedics, where tracheal intubation was deemed the most important [12]. However tracheal intubation may also be viewed as part of the “exclusive body of knowledge” that defines a profession, such that a threat to intubation is a threat to the profession itself [18]. Although the paramedics in our study recognised that other airway management methods are often adequate there was a lack of trust or belief in the utility of supraglottic devices in difficult situations, when airways are soiled or patient extrication is difficult.

It is apparent that in order to successfully manage the airway in the most difficult of circumstances, paramedics should be provided with the best possible training, equipment and support. These issues have been identified previously [13], but there were several suggestions that this is not always the current situation. If airway management can be supported by the use of adjuncts such as supraglottic devices and capnography, and competency can be demonstrated regardless of the frequency of intubation, it remains valuable to encourage the use of a stepwise approach to airway management that includes intubation, since the removal of this skill may act to discourage and disempower UK paramedics.

We have shown that UK paramedics have a wide range of views regarding airway management that are based on evidence and experience, and that although airway management contributes to paramedics’ professional identity, it is not reliant on this. It is apparent that frontline paramedics do not tend to occupy either of the divergent positions of the College of Paramedics or JRCALC [5, 6], but instead are able to use skills, experience and research evidence to form their own opinions. The themes illustrate a profession proud of its skills and contribution to health care and discussion elicited the professionalization agenda. This contemporary debate is emergent in the literature McCann et al., [10], Williams et al., [8], Burford et al., [7]) and illustrates the tensions from within the discipline and from related and influential professions. It is clear that paramedics still have mixed views about their status and associated skills, such as intubation and this is influenced by the educational route taken. If, as McCann et al. [10] discuss, para-medicine is moving towards increasingly professionalized and autonomous practice through its higher level education, professional body, professional journals, state registration and code of ethics, it may seek to hold on to skills, such as intubation, which differentiate it from other professions.

Although this is a small study, attempts were made as far as possible to sample a range of paramedics and therefore a wide range of views. Data saturation was reached and focus groups enabled some deep interrogation of the themes. In light of this research it is suggested that, until a decision is made regarding the effectiveness of alternative airway management methods compared to intubation during OHCA, intubation competency is retained but reviewed to ensure it is supported and provided to a consistently high standard.

Several of the authors of this paper(JBr,MT,SV, JB) are involved in the practice of and research into prehospital airway management. All will hold their own opinions. At the time of this research we used an independent qualitative research, who has since become involved further in prehospital research. We have used robust qualitative methods to ensure that personal opinions are not present.

Qualitative studies are useful in helping to determine future research priorities, and this paper supports numerous others in calling for a randomised control trial in this area [19]. We have demonstrated that paramedics need to be at the centre of any trial investigating a key part of their practice, and that this in turn supports paramedics in the development and consolidation of their profession [7–11, 20].

## Conclusion

We have demonstrated that UK paramedics have a wide range of views regarding airway management, and that these are based on evidence and experience rather than dogma. Airway management contributes to paramedics’ professional identity, but is not reliant on this.
